# Casuistry of oncological diseases in dogs, focusing on bone tumors treated at the Veterinary Hospital of the University of the Republic, Uruguay (2018–2023)

**DOI:** 10.29374/2527-2179.bjvm010325

**Published:** 2026-02-11

**Authors:** Melanie Bazzano Pereira, Rosina Sánchez Solé, Alicia Decuadro Barboza, Carlos Fonseca Alves, Paula Pessina Serdio

**Affiliations:** 1 Departamento de Clínicas y Hospital Veterinario, Facultad de Veterinaria, Universidad de la República (Udelar), Montevideo, Uruguay; 2 Departamento de Cirurgia Veterinária e Reprodução Animal, Faculdade de Medicina Veterinária e Zootecnia, Universidade Estadual Paulista (UNESP), Câmpus de Botucatu, SP, Brazil

**Keywords:** epidemiology, bone neoplasms, dogs, epidemiologia, neoplasias ósseas, cães

## Abstract

Cancer is an important cause of morbidity and mortality in the geriatric canine population; however, there are no updated epidemiological data in Uruguay. In this retrospective study, 963 cases of canine cancer diagnosed between 2018 and 2023 at the Veterinary Hospital Center of the Faculty of Veterinary Medicine (VHC–FVM), Uruguay, were analyzed. Data were collected from medical records and analyzed using descriptive statistics and chi-square tests to assess associations between demographic variables and tumor distribution. Neoplasms of the skin and subcutaneous tissue were the most frequently diagnosed, followed by musculoskeletal and hemolymphatic tumors. A higher proportion of cases were observed in females, most of which were spayed. Skin tumors were more common in intact males, whereas spayed females were more frequently affected by musculoskeletal tumors. The highest number of cases occurred in dogs aged 6 to 10 years, followed by those older than 11 years, and the lowest frequency was observed in dogs aged 0 to 5 years. Among bone tumors, osteosarcoma was the most common type, affecting mainly large breeds and predominantly the appendicular skeleton, particularly the forelimbs. These findings emphasize the importance of considering sex, age, reproductive status, and race in cancer surveillance. From a clinical perspective, such information can guide early diagnosis strategies, improve case follow-up, and support preventive initiatives. In addition, the establishment of local and regional cancer registries is essential for advancing veterinary oncology research and strengthening the understanding of cancer epidemiology in Latin America.

## Resumo

O câncer é uma causa importante de morbidade e mortalidade na população de cães geriátricos; entretanto, não há dados epidemiológicos atualizados no Uruguai. Este estudo retrospectivo analisou 963 casos de neoplasias em cães diagnosticadas entre 2018 e 2023 no Centro Hospitalar Veterinário da Faculdade de Veterinária, Uruguai. Os dados foram obtidos a partir de prontuários clínicos e analisados por meio de estatísticas descritivas e testes do qui-quadrado, com o objetivo de avaliar as associações entre variáveis demográficas e a distribuição dos tumores. As neoplasias de pele e tecido subcutâneo foram as mais frequentemente diagnosticadas, seguidas pelos tumores musculoesqueléticos e hemolinfáticos. Observou-se maior proporção de casos em fêmeas, a maioria delas castradas. Tumores cutâneos foram mais comuns em machos inteiros, enquanto fêmeas castradas foram mais frequentemente acometidas por tumores musculoesqueléticos. O maior número de casos ocorreu em cães com idade entre 6 e 10 anos, seguido por aqueles com mais de 11 anos, sendo a menor frequência observada em cães de 0 a 5 anos. Entre os tumores ósseos, o osteossarcoma foi o tipo mais comum, afetando principalmente cães de raças grandes e localizando-se predominantemente no esqueleto apendicular, especialmente nos membros torácicos. Esses achados ressaltam a importância de considerar o sexo, a idade, o estado reprodutivo e a raça na vigilância oncológica. Do ponto de vista clínico, essas informações podem orientar estratégias de diagnóstico precoce, aprimorar o acompanhamento dos casos e subsidiar iniciativas preventivas. Além disso, o estabelecimento de registros locais e regionais de câncer é essencial para o avanço da pesquisa em oncologia veterinária e para o fortalecimento do entendimento da epidemiologia do câncer na América Latina.

## Introduction

Globally, cancer is the leading cause of death in geriatric dogs, accounting for nearly half of all deaths in this population (Dias Pereira, 2022; Kent et al., 2018; Vascellari et al., 2009). In general, epidemiological data concerning incidence and population frequency are derived primarily from studies conducted at university veterinary hospitals, private institutions, and diagnostic laboratories. Various international databases, such as the Italian Network of Veterinary Oncology Laboratories (NILOV) and the Global Initiative for Veterinary Cancer Surveillance (GIVCS), collect information on cancer in companion animals with the aim of standardizing and coordinating veterinary oncology surveillance systems (Manuali et al., 2019, Pinello et al., 2020; Vascellari et al., 2009).

In South America, epidemiological data on cancer in dogs are still limited, and currently, the only known initiative is the Animal Cancer Registry of São Paulo (Brazil), created in 2013 (Tedardi et al., 2015). In Uruguay**,** information on the epidemiology of canine cancer remains limited, and the only published study has not been updated for more than a decade (Elgue et al., 2012). When attempting to compare results or data, researchers often rely on international studies, which present a challenge, as they may not reflect the demographic or environmental conditions in different populations.

Maintaining updated epidemiological records in animals with cancer makes it possible to identify tumor patterns associated with risk factors, contributing to the identification of canine populations with a greater or lesser probability of developing specific types of tumors (Carvelli et al., 2020; Dobson, 2013; Schwartz et al., 2022). In this context, the generation and updating of local epidemiological data on the basis of demographic variables is critical for determining the risk factors involved in the appearance and progression of cancer in South American dogs (Berthoud et al., 2011; Manuali et al., 2019; Pinello et al., 2020).

Although cancer in dogs has a genetic component (Simpson et al., 2017), it is also influenced by other factors that increase its risk of appearance (Hanahan, 2022). Sex, breed, age, exposure to environmental contaminants, and diet, among other factors, determine cancer susceptibility (Dobson et al., 2002; Kent et al., 2018; Rafalko et al., 2023). With respect to sex, female dogs have a higher overall tumor incidence than males do, a difference largely attributed to mammary cancer (Edmunds et al., 2023; Elgue et al., 2012). Reproductive status has also been identified as a relevant factor in the development of specific cancer types, with increased incidences of hemangiosarcoma, osteosarcoma, lymphoma, and prostatic carcinoma reported in neutered dogs (Bélanger et al., 2017, Grüntzig et al., 2016; Tuohy et al., 2019). Age is also an important predisposing factor for the development of cancer in dogs. The highest frequency of cases of cancer is observed in dogs aged between 6 and 10 years (Edmunds et al., 2023; Fonti et al., 2024; Pinello et al., 2022; Elgue et al., 2012). On the other hand, regarding breeds, it is well established that some have a higher risk of developing specific tumors, such as splenic hemangiosarcoma and multicentric lymphoma in Golden Retrievers (Kent et al., 2018; Urfer & Kaeberlein, 2019); cutaneous mast cell tumors in boxers, French bulldogs, and shar-peis (Catarino et al., 2025); and osteosarcoma in Rottweilers, Great Danes, and Greyhounds (Ru et al., 1998; Tuohy et al., 2019). The latter is the most prevalent primary malignant bone tumor in dogs and represents up to 85% of all bone neoplasms (Anfinsen et al., 2011; Ehrhart et al., 2022). Osteosarcoma affects mainly middle-aged and older dogs (Tuohy et al., 2019), without a clear sex difference. However, a higher incidence of appendiceal presentation has been reported in large and giant breeds (Makielski et al., 2019; Ru et al., 1998; Zapata et al., 2019). In this context, given the variability in the presentation of tumors among canine populations and the limited epidemiological data available locally, this study aimed to provide updated information on the caseload of cancer in dogs, describing the most relevant epidemiological characteristics of the most frequently observed tumor locations, with special emphasis on bone tumors treated at the Veterinary Hospital Center of the Faculty of Veterinary Medicine (VHC–FVM), Uruguay, between 2018 and 2023.

## Materials and methods

All the data were obtained from the files of the VHC–FVM (Uruguay). A descriptive, retrospective study was conducted using the absolute and relative frequencies for categorical variables and was based on a review of 963 medical records of dogs treated at the Oncology Outpatient Clinic from December 2018 to December 2023. Additionally, the relationships between sex, breed, reproductive status and the occurrence of skin/subcutaneous and musculoskeletal tumors were examined.

All dogs with a confirmed cancer diagnosis were included. For each patient, the following data were recorded: Age, sex, reproductive status (intact or neutered), breed, and tumor location and/or affected system. Neoplasms were classified according to the affected system and anatomical location. This categorization was based on the frequency of occurrence and on classifications previously reported in the literature (Grüntzig et al., 2016; Pinello et al., 2022; Elgue et al., 2012). The categories were as follows: 1) skin and subcutaneous tissue; 2) musculoskeletal (ungual, perineal, muscular, and bone tumors); 3) hemolymphatic (splenic, lymph nodal, and hematologic); 4) gastrointestinal (oral, intestinal, rectal, and hepatic); 5) mammary; 6) respiratory (upper respiratory tract and pulmonary); 7) reproductive (penile, vulvovaginal, prostatic, and testicular); 8) endocrine (thyroid, pancreatic, and adrenal); 9) urinary (vesical, renal); and 10) mediastinal. Tumors with a low frequency (fewer than 10 cases) were grouped under the general category “others.” Importantly, the number of cases recorded in the endocrine tumor category may have been underestimated, as most of these patients were treated directly by the Endocrinology Department of our hospital and, therefore, may not have been included in the oncology registry used for this study. With respect to age distribution, patients were grouped into three age ranges: 0–5 years, 6–10 years, and over 11 years, following the criteria previously established in similar studies on canine cancer (Fonti et al., 2024; Rafalko et al., 2023; Elgue et al., 2012). Additionally, 41 canine breeds were identified; breeds with a frequency greater than 20 were maintained as individual categories. Pure breeds with lower representation were grouped under the category “other breeds.” In the musculoskeletal tumor group, we described the main characteristics of patients with bone neoplasms, including race, age, sex, and reproductive status, along with the type of tumor identified by histology or cytology. Those diagnosed with osteosarcoma were grouped into axial or appendicular, indicating that the bone was involved.

The proportion of neoplasms diagnosed (n = 963) at the VHC–FVM, Uruguay, between December 2018 and December 2023 was determined, as well as their distribution according to sex, reproductive status, breed, age group, anatomical location, and affected system. The chi-square test was applied to evaluate associations between sex, reproductive status, and breed and the occurrence of cutaneous and subcutaneous tumors, as well as musculoskeletal neoplasms, using a 95% confidence level. Analyses were performed with SPSS Statistics version 29, and p < 0.05 was considered to indicate statistical significance.

## Results

### General frequency by system and anatomical site

**Figure 1:**
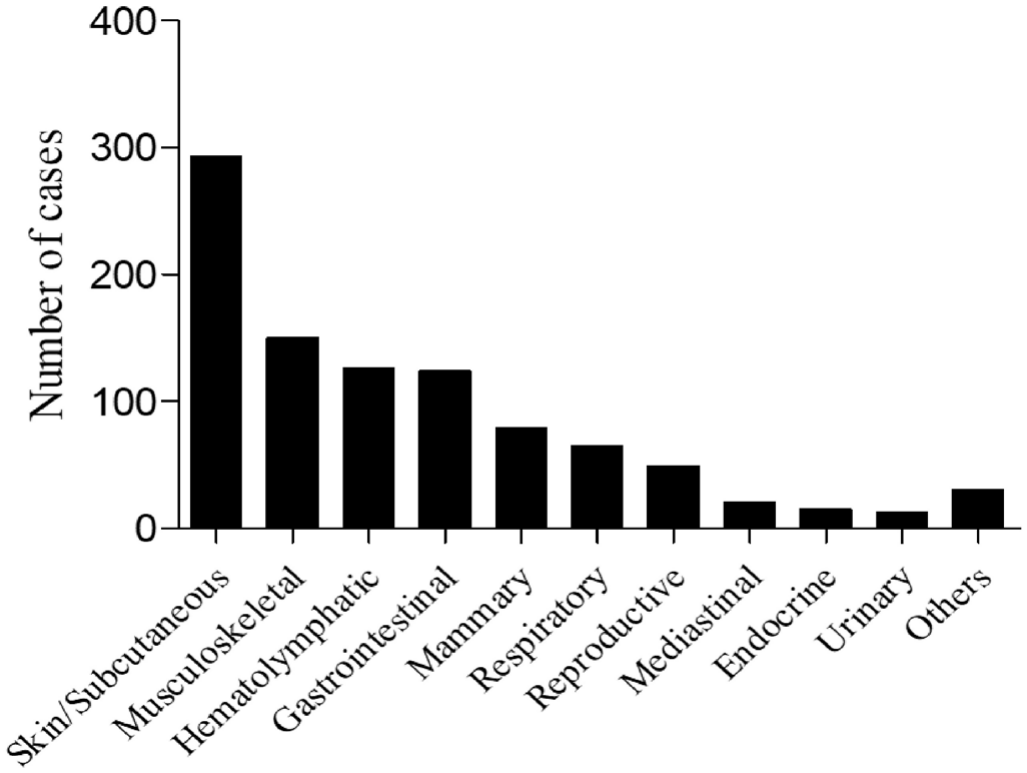
Skin and subcutaneous tumors represent the most frequent neoplasms in dogs: frequency distribution (2018–2023).

During the study period, 963 consultations corresponding to dogs treated at the Oncology Outpatient Clinic of the (VHC–FVM), Uruguay, were recorded. The most frequently diagnosed neoplasms were skin and subcutaneous tissue cancers (293 cases), followed by tumors of the musculoskeletal system (150/963 cases), hemolymphatic neoplasms (126 cases), gastrointestinal neoplasms (124 cases), mammary tumors (79 cases), respiratory tumors (65 cases), reproductive tumors (49 cases), mediastinal tumors (20 cases), endocrine tumors (15 cases), and urinary tumors (12 cases). Finally, the category of “others” included tumors with the lowest frequency, such as cardiac, ophthalmologic, auricular, nervous system, and mesothelial tumors (30 cases total) (Figure 1).

There were 834 cases of canine neoplasms classified according to the affected system and anatomical location. For this analysis, 422 cases were excluded, corresponding to skin and subcutaneous tumors (n = 293), mammary tumors (n = 79), mediastinal tumors (n = 20), and those grouped as “others” (n = 30), because most of these cases lacked precise anatomical location records and/or did not fit into a classification based on affected systems (Table 1).

**Table 1:** Distribution of 541 canine neoplasms by system and anatomical location (2018–2023).

Affected system	Anatomical location				Frequency (n)	% of the total (541)			
Hemolymphatic		Lymph nodal		89			16.5		
		Splenic		33				6.1	
		Hematologic		3				0.5	
					
Musculoskeletal		Nail bed		20				3.7	
		Perineal		34				6.2	
		Cavitary		12				2.2	
		Osseous		59				10.1	
		Musculotendinous		25				4.6	
					
Gastrointestinal		Oral		93				17.1	
		Hepatic		19				3.5	
		Intestinal		9				1.6	
		Rectal		3				0.5	
					
Respiratory		Upper respiratory		50				9.3	
		Pulmonary		15				2.7	
					
Reproductive		Penile		15				2.8	
		Vulvovaginal		21				3.9	
		Testicular		10				1.8	
		Prostatic		3				0.5	
					
Endocrine		Thyroid		9				1.7	
		Pancreatic		3				0.5	
		Adrenal		3				0.5	
					
Urinary		Bladder		11				2.1	
		Renal		1				0.1	

**Figure 2:**
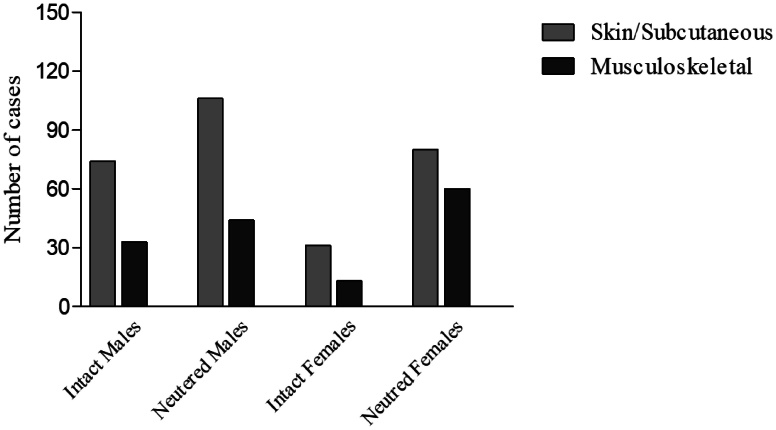
Sex and reproductive status distribution of skin/subcutaneous and musculoskeletal tumors (2018-2023).

Among the total number of dogs with cancer, 550 were females (57.1%), and 413 were males (42.9%). With respect to reproductive status, 71.7% of the females were spayed at the time of consultation, while only 36.5% of the males were neutered. Skin and subcutaneous tumors were more common in males than in females (43.6% [180/413] vs. 20.2% [111/550], p < 0.0001). Reproductive status also significantly affected the frequency of this tumor type: it was higher in intact males than in intact females (41.1% [74/180] vs. 27% [31/111], p < 0.0001). Sex did not affect the frequency of musculoskeletal tumors, with 18.6% (77/413) of males and 13.3% (73/550) of females affected (p = 0.472). However, in terms of reproductive status, spayed females had a significantly greater percentage of this tumor type (60/73, 82%) than neutered males did (33/77, 43%) (p < 0.0001). A descriptive visualization of these distributions is shown in Figure 2.

### Distribution by age

The age group with the greatest number of dogs diagnosed with cancer was 6–10 years (n = 536), followed by those over 11 years (n = 302) and those aged 0–5 years (n = 125). The 6–10-year age group included 57% of the recorded skin and subcutaneous tumors (166/293), 59% of the musculoskeletal tumors (88/150), and 54% of the lymphatic tumors (68/126). The group aged over 11 years included 43.5% with gastrointestinal tumors (54/124), 32% with skin and subcutaneous tumors (93/293), and 30.6% with musculoskeletal tumors (46/150). Conversely, the youngest age group (0–5 years) accounted for 35% of the reproductive tumors (17/49), 26% of the hemolymphatic tumors (33/126), and 21% of the musculoskeletal tumors (31/150).

### Distribution by breed and presentation of skin/subcutaneous and musculoskeletal tumors

Among all the registered cases, 58% of the neoplasms (558/963) occurred in purebred dogs, with a total of 41 breeds recorded. Among these, the highest frequencies were observed in Labrador Retrievers (7.9%, 76/963), followed by Golden Retrievers (6.8% (65/963), Pitbulls (5.9%, 57/963), Poodles (5.1%, 49/963), German Shepherds (3.5%, 34/963), Boxers and Cimarrón Uruguayo (3.3% each, 32/963), and Cockers (3%, 29/963). Crossbred or mixed-breed dogs represented 42% (404/963) of the affected population. There were no significant differences between the mixed-breed and purebred dogs regarding the presentation of skin and subcutaneous tumors (43.3% [127/293] vs. 57% [166/293], p = 0.574). Similarly, musculoskeletal tumors did not significantly differ between purebred and mixed-breed dogs (61.3% [92/150] vs. 38.7% [58/150], p = 0.368).

### Demography and location of bone tumors.

During the study period, a total of 59 cases of canine bone neoplasms were recorded, representing 39.3% of all neoplasms classified within the musculoskeletal group (n = 150). There was no significant difference in the frequency of presentation based on sex, with 29 males (11 neutered and 18 intact) and 28 females (17 spayed and 11 intact) affected. The mean age of the patients was 8 years, ranging from 2 to 15 years. In this context, 56% (33/59) of the cases were registered in the 6–10-year age group, followed by the over-11-year age group (24%, 14/59) and the 0–5-year age group (20%, 12/59).

Among the 59 bone neoplasms, 9 were located in the axial skeleton, and 50 were in the appendicular skeleton. With respect to the recorded histopathological and/or cytological diagnosis, 96% (57/59) were classified as osteosarcomas; the remaining two were diagnosed as one fibrosarcoma and one chondrosarcoma. Among the axial tumors classified as osteosarcomas (n = 8), three were located in the mandible, two were in the maxilla, two were in the frontal bone, and one involved the costal bones. Among the 50 appendicular neoplasms, only one was a fibrosarcoma.

Appendicular osteosarcomas accounted for 49 cases, of which females represented 57% (22 spayed and 6 intact), while males represented 43% (9 neutered and 12 intact) (Table 2). Additionally, of the total number of osteosarcoma cases, 77.6% (38/49) corresponded to purebred dogs, while 22.4% (11/49) were mixed breeds. Within the purebred group, mainly large and giant breeds were identified, with Rottweilers being the most represented (Table 2). The remainder (n = 15) were distributed among other breeds such as Pitbulls, Golden Retrievers, Labradors, Greyhounds, Great Danes, Dobermans, Brazilian Mastiffs, Cocker Spaniels, Kelpies, Weimaraners, and Siberian Huskies.

**Table 2:** Demographic characteristics of 49 dogs with appendicular osteosarcoma (2018–2023).

Category	Variable	n	%
	Female	28	57.1
	Spayed	22	78.5
Sex	Intact	6	21.5
			
	Male	21	42.9
	Neutered	9	43.0
	Intact	12	57.0
			
Age	Mean (years)	8	-
	Age range (years)	2–15	-
			
			
			
	Mixed breed	11	22.4
			
Breed	Purebred	38	77.6
	Rottweiler	7	18.4
	Neapolitan Mastiff	5	13.1
	Saint Bernard	4	10.5
	German Shepherd	4	10.5
	Cimarrón Uruguayo	3	7.9
	Other breeds	15	39.6

**Figure 3:**
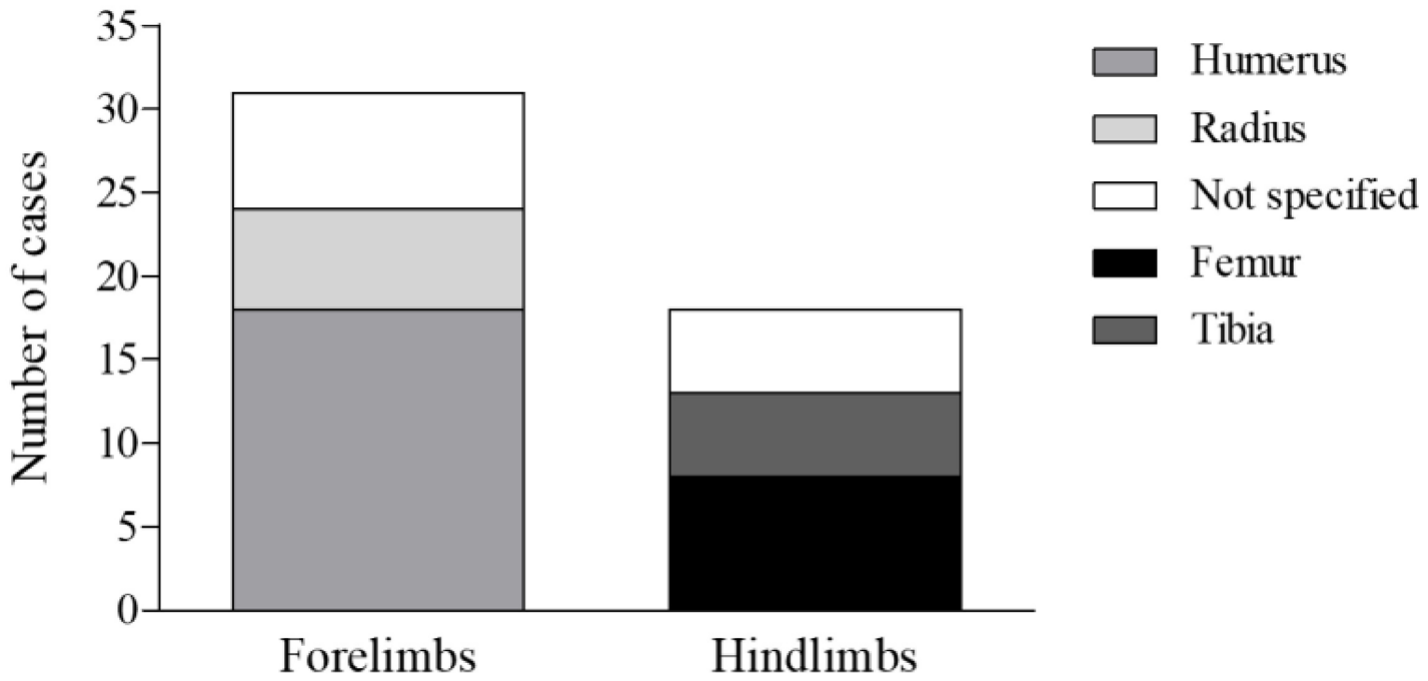
The forelimbs are the most common site of canine appendicular osteosarcoma: distribution by bone (2018-2023).

Among the total appendicular skeleton tumors, there was a higher frequency of presentation in the forelimbs (63%, 31/49), with the humerus being the most affected bone (n = 18), followed by the radius (n = 6); in 7 cases, this information was not available. Moreover, the remaining 37% (18/49) of the tumors were located on the hindlimbs, with the femur (n = 8) and tibia (n = 5) being the most frequently involved bones; in 5 cases, the specific bone involved was not specified (Figure 3).

## Discussion

This study provides updated information on the demographics and frequency of tumor presentation in dogs treated at the Teaching Hospital of the VHC–FVM, Uruguay, between 2018 and 2023. With respect to tumor location in the studied population, skin and subcutaneous tissue neoplasms were the most frequent, which is consistent with reports from international and regional registries, as these tumors represent one of the main reasons for oncological consultation in dogs (Manuali et al., 2019; Pinello et al., 2022; Tedardi et al., 2015; Vascellari et al., 2009). However, our results differ from those of a previous study conducted at the same institution, where mammary tumors were the most frequent (Elgue et al., 2012). In our study, breast neoplasms were fifth in frequency; this difference may be attributed to the increase in different dog population control initiatives and changes in referral patterns and access to oncology services over the past decade. In this context, it is important to consider the progressive cultural change around responsible pet ownership in Uruguay, promoted since 2009 by the Commission on Responsible Ownership and Animal Welfare (COTRYBA). These efforts have been reinforced by massive sterilization and microchip campaigns organized by various departmental governments and municipalities, aimed at preventing zoonoses and controlling animal populations, supported by the National Institute of Animal Welfare (INBA) since 2018. These campaigns have promoted early sterilization, which may have influenced both the incidence of certain oncological diseases and the profile of animals that access specialized care.

In our study population, there was a higher frequency of cases in females than in males, which is consistent with reports in the literature (Fonti et al., 2024; Schwartz et al., 2022). Some authors have attributed this higher frequency in females to the high prevalence of mammary gland tumors (Hart et al., 2020; Manuali et al., 2019; Elgue et al., 2012). However, in our study, mammary tumors represented only the fifth most frequent group, suggesting that this female predominance is explained by other tumor types, particularly skin and subcutaneous neoplasms. In terms of reproductive status, a greater percentage of females were spayed (71.7%) compared with neutered males (36.5%). This pattern aligns with observations in other population studies (Dias Pereira, 2022; Carvelli et al., 2020), which highlight a higher frequency of preventive sterilization in females, which is commonly associated with population-control campaigns. In South American countries, the prevalence of castrated dogs in the general population remains relatively low. This is likely influenced by cultural factors, with non-castrated dogs still constituting the majority in many developing countries (Berthoud et al., 2011). Moreover, spayed females in our study presented the highest frequency of musculoskeletal tumors, which is consistent with previously published results suggesting that spaying in females is linked to certain cancer types, including osteosarcoma (Kent et al., 2018; Grüntzig et al., 2016). From an oncological perspective, although early sterilization reduces the risk of some tumors, such as mammary, testicular, and perineal cancers (Urfer & Kaeberlein, 2019), it has also been associated with an increased risk and incidence of others, such as hemangiosarcoma, lymphoma, and bladder cancer, particularly in certain breeds (Dobson, 2013; Hart et al., 2020). Overall, these findings emphasize the need for an individualized approach in the practice of canine sterilization, considering both its preventive effects and its possible effects on the risk of tumor development.

With respect to age, the highest concentration of neoplasms was observed in animals aged 6–10 years, a finding that is consistent with previous publications identifying this age range as the highest risk period for dogs (Dobson et al., 2002; Fonti et al., 2024; Elgue et al., 2012). This predominance in middle-aged and older individuals can be explained by the natural aging process, during which genetic alterations accumulate and the immune system’s ability to eliminate abnormal cells declines (Hanahan, 2022). Additionally, advances in preventive medicine, nutrition, and care have contributed to increased longevity in the canine population, increasing the likelihood of neoplasm development throughout life (Kent et al., 2018; Schwartz et al., 2022). On the other hand, although cancer was less frequent in young animals in our study, its presence warrants careful interpretation. Moreover, in animals aged 0–5 years, the most frequent tumors—excluding those of the reproductive system—were hemolymphatic and musculoskeletal neoplasms. In young dogs, certain neoplasms, such as lymphoma and osteosarcoma, have a significant genetic influence, and some breeds have specific predispositions for them (Dobson, 2013; Kent et al., 2018; Tuohy et al., 2019). Unlike oncological processes related to aging, juvenile cases result not only from cumulative cellular damage but also from hereditary alterations that predispose patients to early tumor development (Pinello et al., 2022). A higher incidence of osteosarcoma has been identified in large and giant breeds such as Rottweilers and Great Danes, whereas lymphoma is highly prevalent in Golden Retrievers and Boxers (Dobson, 2013; Rafalko et al., 2023). This evidence highlights the need to incorporate a differential approach based on age and race for the oncological surveillance of our patients.

In terms of the canine population with bone tumors, osteosarcoma was the most common primary bone tumor, which is consistent with the findings of studies from other countries (Simpson et al., 2017; Tuohy et al., 2019). There was a similar sex distribution of osteosarcoma in our study, which aligns with studies demonstrating no clear sex predisposition for osteosarcoma occurrence (Tuohy et al., 2019). Additionally, our findings coincide with reports in the literature indicating a higher proportion of neutered animals among osteosarcoma cases (Bélanger et al., 2017; Ru et al., 1998; Tuohy et al., 2019). The absence of gonadal hormones may double the risk of developing osteosarcoma in dogs (Grüntzig et al., 2016). Moreover, neutering before 1 year of age in breeds predisposed to develop osteosarcoma, such as Rottweilers, Great Danes, German Shepherds, and Golden Retrievers, is an important risk factor for disease development (Grüntzig et al., 2016; Hart et al., 2020; Ru et al., 1998). However, this study did not evaluate the correlation between reproductive status and disease presentation; therefore, we could not determine whether neutered dogs (63.3%) in the studied population had an increased risk of developing osteosarcoma.

In our study, the mean age of the dogs with osteosarcoma was 8 years. This disease primarily affects middle-aged and older dogs, whose ages range from 6 to 9 years at the time of diagnosis (Makielski et al., 2019; Simpson et al., 2017). However, osteosarcoma may occur less frequently in young dogs aged 1.5 to 3 years, and the disease is described as having a bimodal age distribution (Edmunds et al., 2021; Ehrhart et al., 2022). This bimodal distribution was not observed in this study; however, tumors were more frequent in dogs aged 6 to 10 years, which is in agreement with population studies in which osteosarcoma is characterized as a disease of adult and senile dogs (Anfinsen et al., 2011; Makielski et al., 2019; Tuohy et al., 2019). This study revealed that 77% of the osteosarcoma cases belonged to a defined breed, with Rottweilers being the most represented (18.4%). These findings align with several investigations identifying high-risk defined breeds and linking body size and height as predisposing factors for osteosarcoma development (Edmunds et al., 2021; Ru et al., 1998). Notably, an increased predisposition has been described in Rottweilers, Irish Wolfhounds, Greyhounds, Saint Bernards, Great Danes, and Golden Retrievers, among others, which has been largely attributed to genetic factors (Simpson et al., 2017; Tuohy et al., 2019; Zapata et al., 2019). Moreover, it has been proposed that selective breeding aimed at increasing body size in certain dog breeds contributes to the concentration of alleles associated with osteosarcoma development, explaining why fewer than 5% of osteosarcoma cases occur in dogs weighing less than 15 kg (Makielski et al., 2019; Zapata et al., 2019).

With respect to clinical presentation, our findings align with the literature reporting a predilection of appendicular osteosarcoma for the forelimbs compared with the hindlimbs (Dobson, 2013; Makielski et al., 2019). In our case series, 63% of cases were localized to the forelimbs, with the humerus being the most frequently affected bone. This distribution also concurs with previous studies that have shown a higher incidence in weight-bearing bones such as the humerus and radius (Anfinsen et al., 2011; Tuohy et al., 2019). This pattern may be explained by the fact that the forelimbs bear more than 60% of their body weight; thus, they are exposed to multiple and repeated microtraumas, which could predispose them to neoplastic cellular transformation (Hanahan, 2022; Ru et al., 1998). Moreover, osteosarcoma presentation in the humerus is a poor prognostic factor, as it is associated with high rates of pulmonary metastasis and short survival times (Ehrhart et al., 2022). These findings highlight the importance of anatomical and biomechanical factors in the development and clinical behavior of appendiceal osteosarcoma in dogs.

## Conclusion

During the 2018–2023 study period at the VHC–FVM, Uruguay, skin and subcutaneous neoplasms were the most frequent tumors, followed by musculoskeletal ones. Sex and reproductive status influenced tumor occurrence; while females represented most oncological cases, skin and subcutaneous tumors were more common in intact males, and spayed females presented a greater proportion of musculoskeletal neoplasms. The mean age at diagnosis was 8 years, with most cases occurring between 6 and 10 years, reflecting the increased cancer risk with advancing age. Given the observed associations between spaying and specific tumor types, the decision to neuter should be carefully individualized, with preventive benefits against potential oncologic risks for each sex, breed, and age group weighed.

With respect to bone neoplasms, osteosarcoma was the most frequently observed tumor type, primarily affecting older adult dogs of defined breeds. Notably, osteosarcoma was more frequently localized in the appendicular skeleton, with the forelimbs being the most affected, particularly the humerus. Thus, we reinforce the importance of epidemiological and clinical factors in the early detection of this disease in canines.
